# Machine learning models for predicting the risk of depressive symptoms in Chinese college students

**DOI:** 10.3389/fpsyt.2025.1648585

**Published:** 2025-08-05

**Authors:** Chengfu Yu, Xiangxuan Kong, Weijie Yu, Xingcan Ni, Jing Chen, Xiaoyan Liao

**Affiliations:** ^1^ Department of Psychology/Research Center of Adolescent Psychology and Behavior, School of Education, Guangzhou University, Guangzhou, Guangdong, China; ^2^ School of Psychology, South China Normal University, Guangzhou, Guangdong, China

**Keywords:** machine learning, depressive symptoms, risk and protective factors, college students, random forest

## Abstract

**Introduction:**

Depression is highly prevalent among college students, and accurately identifying risk factors is essential for timely intervention. Given the limitations of traditional linear models in managing high-dimensional data, this study employed machine learning techniques to predict depressive symptoms.

**Method:**

Data were collected from 1,635 Chinese college students and included 38 sociodemographic, psychological, and social variables. Four machine- learning algorithms, Random Forest, XGBoost, LightGBM, and Support Vector Machine, were evaluated.

**Results:**

Results showed that the Random Forest model achieved the highest discriminant performance with an AUC of 0.87 and an accuracy of 0.79, and identified key predictors such as sleep disturbance, perceived stress, experiential avoidance, and self-criticism. SHapley Additive exPlanations analysis further revealed that deteriorating sleep quality and heightened stress levels significantly increased the risk of depressive symptoms.

**Discussion:**

These findings validate the effectiveness of Random Forest in capturing complex data interactions and offer actionable insights for targeted mental health interventions. Future studies should improve generalizability by incorporating more diverse samples and physiological biomarkers.

## Introduction

Depression is a significant psychological and public health concern, imposing a substantial burden on global health systems and contributing to considerable socioeconomic losses (1). Persistent depressive symptoms adversely affect individuals’ emotional well-being, social functioning, and cognitive development (2–4). Of further note, they can elevate the risk of progressing to a major depressive disorder (5) and developing suicidal ideation (6). Among college students, depressive symptoms are particularly prevalent. A recent study in China found that 24.5% of college students reported experiencing such symptoms (7). Given the high prevalence and potential long-term consequences, identifying the predictors of depressive symptoms in this population is essential to support early detection, effective monitoring, and timely intervention.

According to ecological systems theory (8), individual development is shaped by the dynamic interaction between the person and multiple surrounding social systems. Research has shown that the onset of depressive symptoms among college students is influenced by a wide range of factors. In addition to demographic characteristics (9), individual psychological and behavioral factors, encompassing emotional and cognitive dimensions, are significantly linked to depressive symptoms (10–13). Furthermore, susceptibility traits such as neuroticism (14) and various psychopathological symptoms, including alexithymia, Internet addiction, and mobile phone addiction, have also been identified as significant correlates (14–16). Family-level variables are particularly salient when considering the broader social-contextual environment of college students. Well-established risk factors include childhood trauma and maladaptive parenting styles (17, 18). Additionally, experiences of family dysfunction, cyberbullying victimization, and exposure to stressful life events are consistently linked to increased vulnerability to depressive symptoms in this population (18, 19).

Accurately identifying individuals with depressive symptoms remains a significant challenge, as precise prediction requires integrating a variety of individual and social-contextual factors. However, to ensure stability and reproducibility, traditional linear models must limit the number of predictors relative to the sample size, and the included variables should not be highly correlated. These constraints reduce the ability of traditional multiple regression models to effectively identify potential predictors, leading to lower predictive power (20). Machine learning (ML), a data-driven branch of artificial intelligence, can flexibly handle high-dimensional datasets and capture the simultaneous effects of all relevant predictors more accurately, often outperforming traditional stepwise analysis methods (21). Consequently, researchers have begun to apply ML techniques to the early identification of depression and depressive symptoms. For instance, Luo et al. (22) employed a Random Forest classifier to analyze factors associated with depression risk, including socioeconomic conditions, demographic characteristics, family history of mental health, behavioral and lifestyle factors, and physical and mental health indicators. They found that psychological factors, such as suicidal ideation, anxiety, and sleep quality, showed the strongest associations. Gohari et al. (9) likewise used a Random Forest algorithm to predict depression among Canadian adolescents, identifying key predictors such as home life, school connectedness, mental health measures (anxiety symptoms, emotional dysregulation, and flourishing), gender, and sleep duration. However, these studies primarily focused on predicators from limited domains. Furthermore, although different ML algorithms offer distinct strengths, most research to date has relied on a single algorithm. Therefore, it is necessary to compare multiple ML models to develop the most effective approach for predicting depressive symptoms among college students.

In summary, this study considers a range of sociodemographic, individual (including personal traits, psychopathological symptoms, and emotional, cognitive, and behavioral factors), and social contextual variables, aiming to develop an optimal model for predicting depressive symptoms among college students by applying and comparing multiple advanced ML algorithms. Furthermore, this study aims to accurately identify key risk and protective factors that significantly influence depressive symptoms in this population. These findings may offer a more precise and accessible method for predicting depressive symptoms, enabling schools, parents, and healthcare professionals to support early detection and implement targeted interventions.

## Method

### Participants and procedure

A total of 2115 students from six Chinese universities in central and south China participated in the study. All data were collected offline using self-report questionnaires. The questionnaires were distributed in university classrooms by graduate students and psychology professors, accompanied by identical verbal and written instructions. After excluding questionnaires with non-standard responses (e.g., implausible age values and missing critical data such as demographics), 1,635 valid questionnaires were retained for analysis. The sample consisted of 558 males (34.12%) and 1,077 females (65.88%) aged 17–24 years (*M_age_* = 18.93 years, *SD* = 1.23 years).

### Measure

#### Depressive symptoms

The Patient Health Questionnaire-9 (PHQ-9) (23) was used to assess depressive symptoms. The PHQ-9 was originally measured from 0 (*not at all*) to 3 (*nearly every day*) with a possible total score of 0–27. Since most of our scales here start with 1, and to be as consistent as possible, the PHQ-9 in this article uses a four-point scoring system ranging from 1 (*not at all*) to 4 (*nearly every day*), with total scores ranging from 9 to 36. Scores of 14–18 indicate mild depression, 19–23 indicate moderate depression, 24–28 denote moderate-to-severe depression, and 29–36 suggest severe depression. An example item is “Feeling down, depressed, or hopeless.” In this study, scores above 14 were considered indicative of depressive symptoms. The Cronbach’s alpha for the scale in this sample was 0.88.

#### Socio-demographic variables

Participants reported sociodemographic information, including age, gender, only child status, place of origin, family economic situation, and family structure. These variables were included as potential predictors in the analysis.

#### Psychological, psychiatric, and social factors

This dataset included the following correlates of depressive symptoms: alexithymia (24), sleep disturbance (25), suicidal behaviors (26), self-injury (27), smartphone addiction (28), social media addiction (29), self-control (30, 31), emotion regulation (32, 33), growth mindset (34), self-compassion (35, 36), meaning in life (37), fear of negative evaluation (38, 39), self-criticism (40), basic psychological needs frustration (41), impulsivity (30, 42), experiential avoidance (43, 44), intolerance of uncertainty (45, 46), Big Five personality traits (47), childhood trauma (48), bullying victimization (49), parental emotion socialization (50), and perceived stress (51, 52). Additional details on the assessment of each variable are provided in the **Supplementary Text**.

### Statistical analysis

Descriptive statistical analyses were first conducted using SPSS 26.0. ML modeling was then performed using the Scikit-learn library in Python (53). To enhance the generalization ability of the models, data preprocessing was conducted using the StandardScaler method, which normalized all features to have a mean of zero and a standard deviation of one. This approach reduced data bias arising from differences in measurement scales. The outcome variable was whether participants reported mild to severe depressive symptoms. We used an independent-samples *t*-test and a chi-square test for feature selection. Thirty-eight variables associated with depressive symptoms were selected. These thirty-eight variables were used as predictors, including sociodemographic characteristics, emotional disorder states, and coping styles.

Four ML algorithms were employed to construct separate risk prediction models: Random Forest, eXtreme Gradient Boosting (XGBoost), the Light Gradient Boosting Machine (LightGBM), and Support Vector Machine (SVM) (54). Each algorithm was selected for its ability to handle imbalanced data and enhance model generalizability. Random Forest improves model stability and predictive accuracy by aggregating multiple decision trees and randomly selecting feature subsets at each split, thereby reducing the risk of overfitting (55). XGBoost, based on gradient-boosted decision trees, optimizes its objective function with regularization terms, demonstrating high efficiency and accuracy in handling complex datasets (56). LightGBM, similar to XGBoost, adopts a leaf-wise splitting strategy to improve computational efficiency, although it may be more prone to overfitting in certain scenarios (57). SVM identifies the optimal hyperplane for classification and demonstrates strong generalization capabilities, particularly in high-dimensional and small-sample datasets (58). To evaluate model stability and reliability, this study employed 10-fold cross-validation to minimize sample bias (59, 60). For each fold following data partitioning into training and test subsets, SMOTE was employed on the training data. The SMOTE object, initialized with a random state, used the “fit_resample” method to synthesize minority class instances, creating a balanced training set. This augmented dataset was subsequently used to train the Random Forest classifier (61). By generating synthetic samples for underrepresented classes, SMOTE enhances classifier efficacy, especially in classification tasks (62). The models were then trained on each cross-validation training set and evaluated on the corresponding validation set. Multiple performance metrics were calculated (63), including precision, F1-score, accuracy, recall, area under the receiver operating characteristic (ROC) curve (AUC), and the average of each metric across all folds.

To identify the most critical features for depression prediction, this study analyzed feature importance within the ML models and visualized the results using horizontal bar charts. In addition, SHapley Additive exPlanations (SHAP) values were used as a feature importance metric to interpret model predictions (64). As model complexity increases, particularly in ensemble and deep learning models, prediction accuracy tends to improve while interpretability declines. SHAP values, derived from Shapley’s value theory in game theory, quantify feature importance by computing the average marginal contribution of each feature to a given prediction. These values satisfy key properties such as fairness, uniqueness, and efficiency and provide both baseline and individual feature contributions for each prediction. The sum of the feature contributions equals the difference between the model output and the baseline, allowing users to understand the model’s logic and the actual impact of each input variable (65). Accordingly, this study calculated and visualized SHAP values for all models.

## Results

### Descriptive statistics

The study analyzed 1,635 valid responses, with 827 participants (50.5%) meeting criteria for depressive symptoms. **Table 1** presents descriptive statistics and general characteristics of the study variables. Statistical analyses revealed significant differences in nearly all measured variables between participants exhibiting depressive symptoms and those without, except for gender, only child status, and family structure (see **Tables 1**, **2**).

**Table 1:** Descriptive statistics and general characteristics of the study variables.

Variable	Overall (*n* = 1635)	Depressive symptoms		*t*	*p*
	*M* (*SD*)	No (*n* = 808) *M* (*SD*)	Yes (*n* = 827) *M* (*SD*)		
Alexithymia	19.39 (6.63)	16.87 (6.33)	21.85 (5.97)	–16.34	<0.001
Sleep disturbance	4.93 (1.82)	4.02 (1.25)	5.83 (1.86)	–23.08	<0.001
Suicidal ideation	0.13 (0.34)	0.06 (0.24)	0.21 (0.40)	–8.84	<0.001
Suicide plan	0.04 (0.19)	0.01 (0.10)	0.07 (0.24)	–5.78	<0.001
Suicide attempt	0.02 (0.12)	0.002 (0.05)	0.03 (0.17)	–4.47	<0.001
Non-suicidal self-injury	0.09 (0.29)	0.04 (0.18)	0.15 (0.35)	–7.99	<0.001
Smartphone usage time	4.71 (1.18)	4.58 (1.17)	4.84 (1.18)	–4.49	<0.001
Pre-sleep smartphone usage	4.22 (1.44)	4.02 (1.39)	4.41 (1.47)	–5.53	<0.001
Smartphone addiction	33.42 (9.00)	30.86 (8.88)	35.92 (8.40)	–11.84	<0.001
Social media addiction	14.46 (4.22)	13.40 (3.98)	15.48 (4.19)	–10.27	<0.001
Self-control	21.14 (3.89)	22.44 (3.99)	19.87 (3.33)	14.09	<0.001
Emotion regulation	44.25 (7.52)	43.73 (7.43)	44.76 (7.58)	–2.76	0.007
Growth mindset	20.44 (5.46)	21.44 (5.72)	19.46 (5.00)	7.43	<0.001
Self-compassion	40.03 (6.10)	42.18 (5.93)	37.93 (5.51)	14.97	<0.001
Meaning in life	46.57 (9.64)	48.75 (9.94)	44.44 (8.84)	9.25	<0.001
Fear of negative evaluation	38.05 (8.29)	35.39 (8.12)	40.65 (7.60)	–13.49	<0.001
Self-criticism	25.37 (6.78)	22.17 (6.30)	28.50 (5.69)	–21.29	<0.001
Basic psycho- logical needs frustration	30.31 (9.22)	25.99 (8.46)	34.52 (7.88)	–21.07	<0.001
Impulsivity	18.07 (3.67)	16.93 (3.72)	19.19 (3.25)	–13.03	<0.001
Experiential avoidance	19.19 (8.69)	15.10 (6.98)	23.18 (8.34)	–21.24	<0.001
Intolerance of uncertainty	35.87 (8.52)	32.69 (8.30)	38.99 (7.52)	–16.06	<0.001
Big five personality traits					
1. Extraversion	10.23 (3.19)	10.75 (3.38)	9.72 (2.90)	6.55	<0.001
2. Neuroticism	10.04 (3.18)	8.75 (3.02)	11.31 (2.80)	–17.75	<0.001
3. Conscientiousness	11.56 (2.63)	11.98 (2.66)	11.15 (2.54)	6.50	<0.001
4. Agreeableness	12.09 (2.90)	12.53 (2.84)	11.67 (2.89)	6.05	<0.001
5. Openness to experience	10.53 (3.18)	11.01 (3.20)	10.06 (3.08)	6.14	<0.001
Childhood trauma	39.32 (10.63)	36.55 (9.10)	42.02 (11.31)	–10.77	<0.001
Bullying victimization	0.64 (1.56)	0.35 (1.15)	0.92 (1.83)	–7.46	<0.001
Parental emotion socialization(angry)	28.49 (8.09)	26.79 (7.88)	30.16 (7.93)	–8.58	<0.001
Parental emotion socialization(fear)	25.26 (7.47)	23.65 (7.21)	26.83 (7.40)	–8.79	<0.001
Parental emotion socialization (Sad)	26.07 (7.34)	24.48 (7.01)	27.62 (7.34)	–8.84	<0.001
Perceived stress	40.23 (7.22)	36.75 (6.74)	43.62 (5.95)	–21.80	<0.001

**Table 2:** Descriptive statistics and general characteristics for the socio-demographic variables.

Variable	Overall (*n* = 1635)	Depressive symptoms		*t/χ^2^*	*p*
	*M* (*SD*)/*n* (%)	No (*n* = 808) *M* (*SD*)/*n* (%)	Yes (*n* = 827) *M* (*SD*)/*n* (%)		
Age	18.93 (1.23)	18.86 (1.16)	19.00 (1.29)	–2.19	0.029
Gender					
Boy	558 (34.1%)	288 (35.6%)	270 (32.6%)	1.63	0.202
Girl	1077 (65.9%)	520 (64.4%)	557 (67.4%)		
Only child					
Yes	248 (15.2%)	132 (16.3%)	116 (14.0%)	1.69	0.193
No	1387 (84.8%)	676 (83.7%)	711 (86.0%)		
Origin					
Rural	907 (55.4%)	435 (53.8%)	472 (57.0%)	8.11	0.017
Town	367 (22.4%)	171 (21.2%)	196 (23.7%)		
City	361 (22.2%)	202 (25%)	159 (19.3%)		
Family structure					
Two-parent family	1447 (88.5%)	729 (90.2%)	718 (86.8%)	5.10	0.164
Single-parent family	120 (7.3%)	49 (6.0%)	71 (8.6%)		
Blended family	43 (2.6%)	20 (2.5%)	23 (2.8%)		
Other family types	25 (1.6%)	10 (1.3%)	15 (1.8%)		
Family economic situation					
Very poor	40 (2.4%)	19 (2.3%)	21 (2.5%)	9.26	0.005
poor	330 (20.2%)	143 (17.7%)	187 (22.6%)		
Average	1112 (68.0%)	558 (69.0%)	554 (67.0%)		
Good	138 (8.5%)	79 (9.8%)	59 (7.1%)		
Excellent	15 (0.9%)	9 (1.2%)	6 (0.8%)		

### Model performance

**Figure 1:**
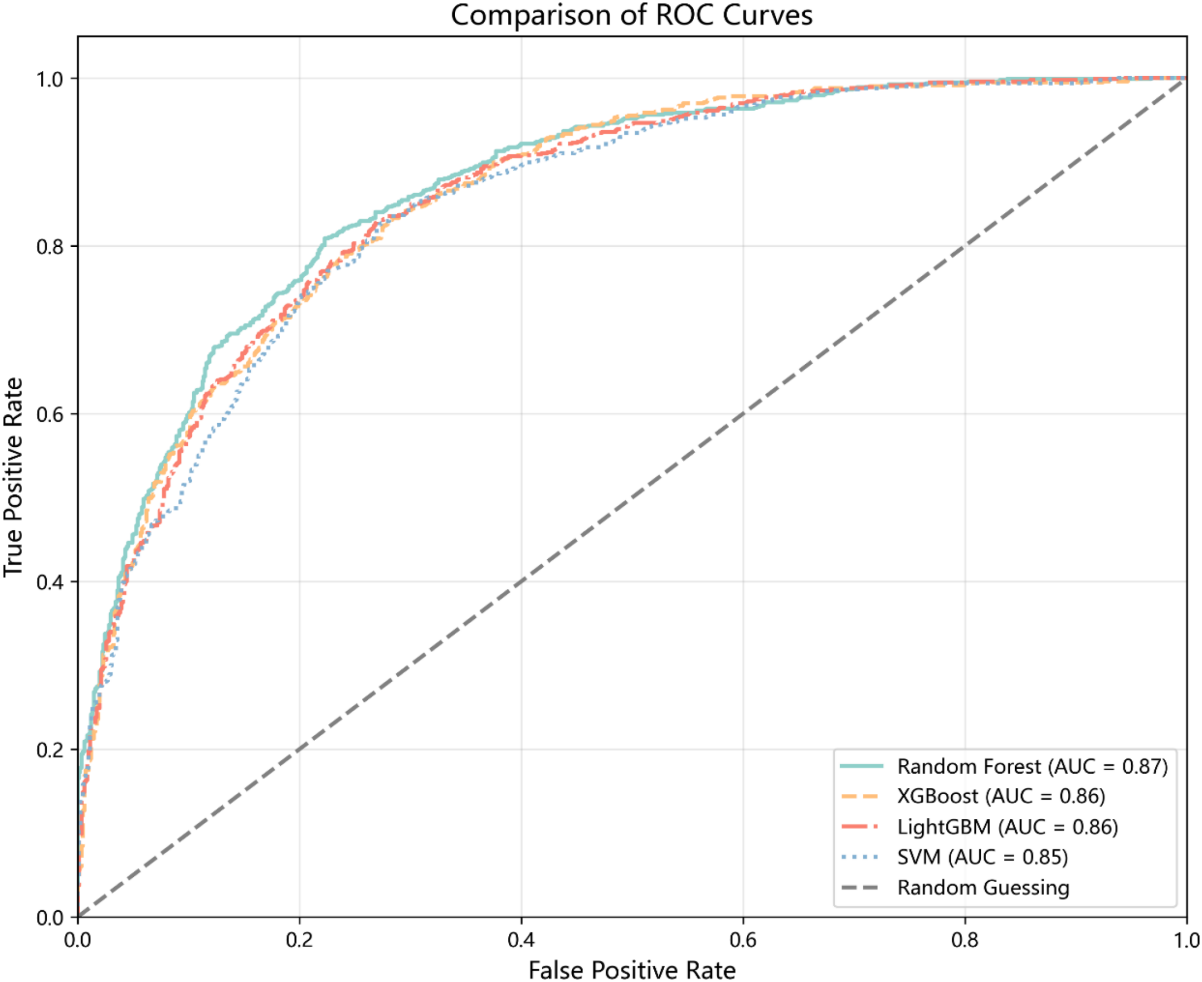
ROC curves for all models.

**Figure 2:**
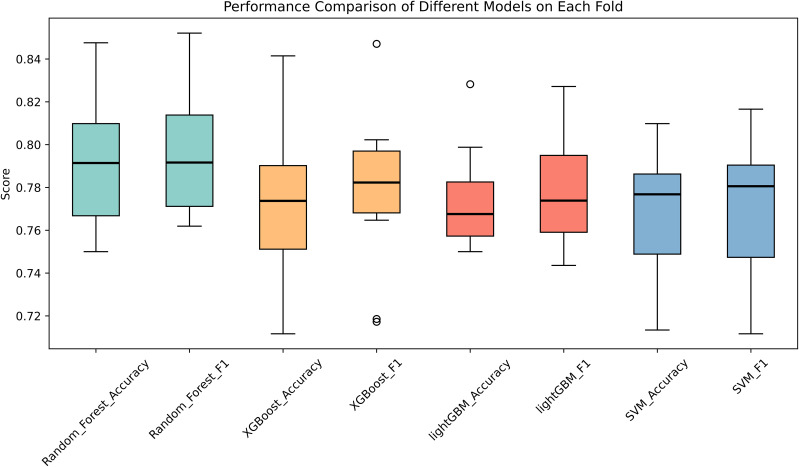
The performance comparison of the four models across each fold.

The ROC curve is a graphical tool used to evaluate the performance of binary classifiers. It illustrates classifier performance across all possible thresholds, allowing assessment of the model’s ability to distinguish between classes regardless of a specific cutoff point. A curve closer to the top-left of the chart indicates better classification performance. A higher AUC value reflects a stronger separation between positive and negative classes. **Figure 1** presents the ROC curves for all four models, each achieving an AUC above 85%, demonstrating strong predictive performance. **Figure 2** illustrates the performance comparison of the four models across each fold, providing a comprehensive view of their respective metrics.

**Table 3** presents the AUC score, accuracy, precision, and specificity of each ML model across all folds of cross-validation. The Random Forest model outperformed the others across all metrics.

**Table 3:** Machine learning models performances for depressive symptoms.

ML model	Accuracy	Precision	Sensitivity	F1 score	AUC score
Random Forest	0.7908	0.7878	0.8041	0.7956	0.8704
LightGBM	0.7730	0.7682	0.7932	0.7785	0.8619
XGBoost	0.7743	0.7737	0.7871	0.7787	0.8594
SVM	0.7688	0.7695	0.7763	0.7721	0.8505

### Variable importance

**Figure 3:**
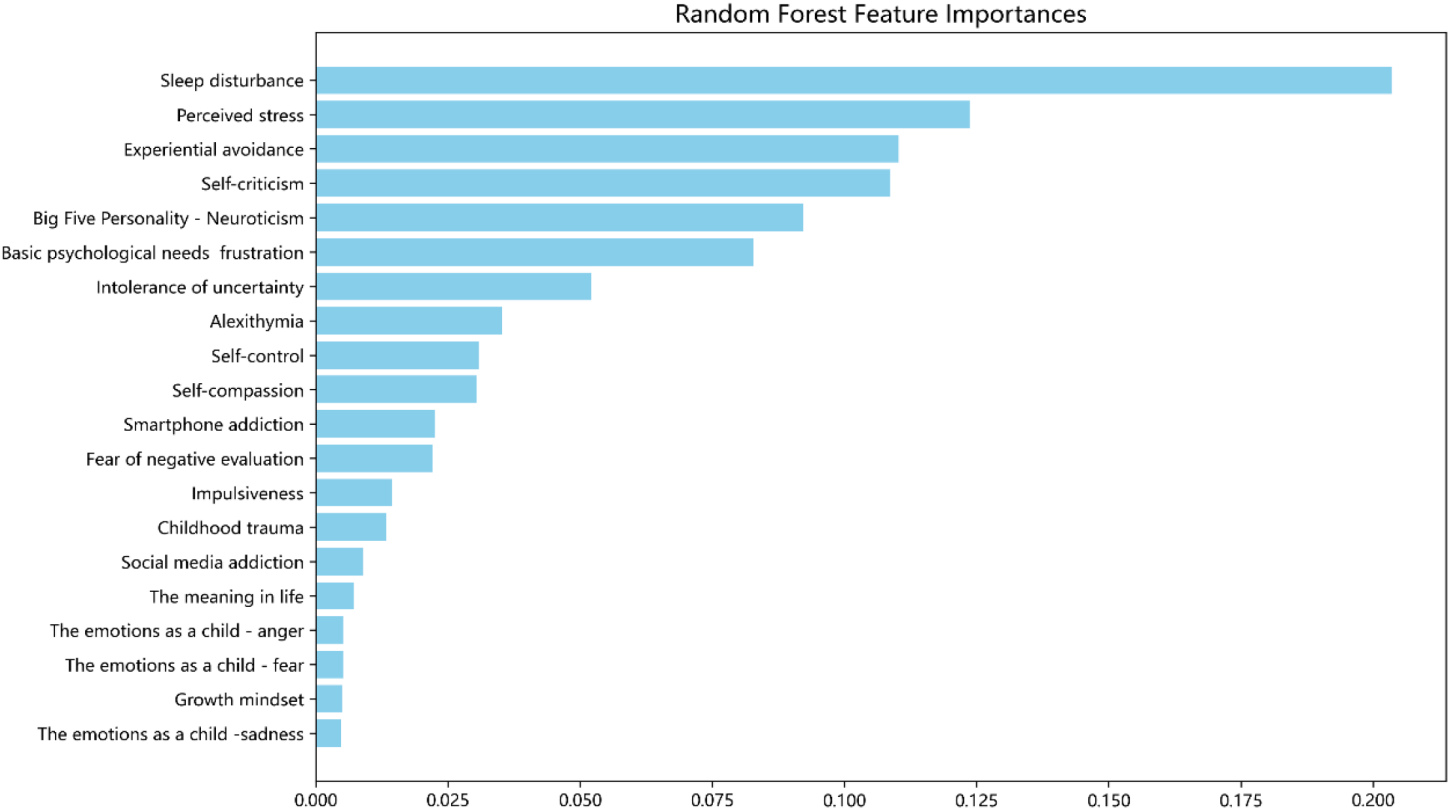
Feature importance plot of the Random Forest, which depicts the importance of each variate in the development of the model.

To visualize the impact of each variable on depressive symptoms more intuitively, we employed the built-in feature importance method to interpret the variable importance in the best-performing Random Forest model. The resulting feature importance plot (**Figure 3**) displays the top 20 most important variables. The most important predictor was sleep disturbance (importance score = 0.17), followed by perceived stress (importance score = 0.11) and experiential avoidance (importance score = 0.09).

**Figure 4:**
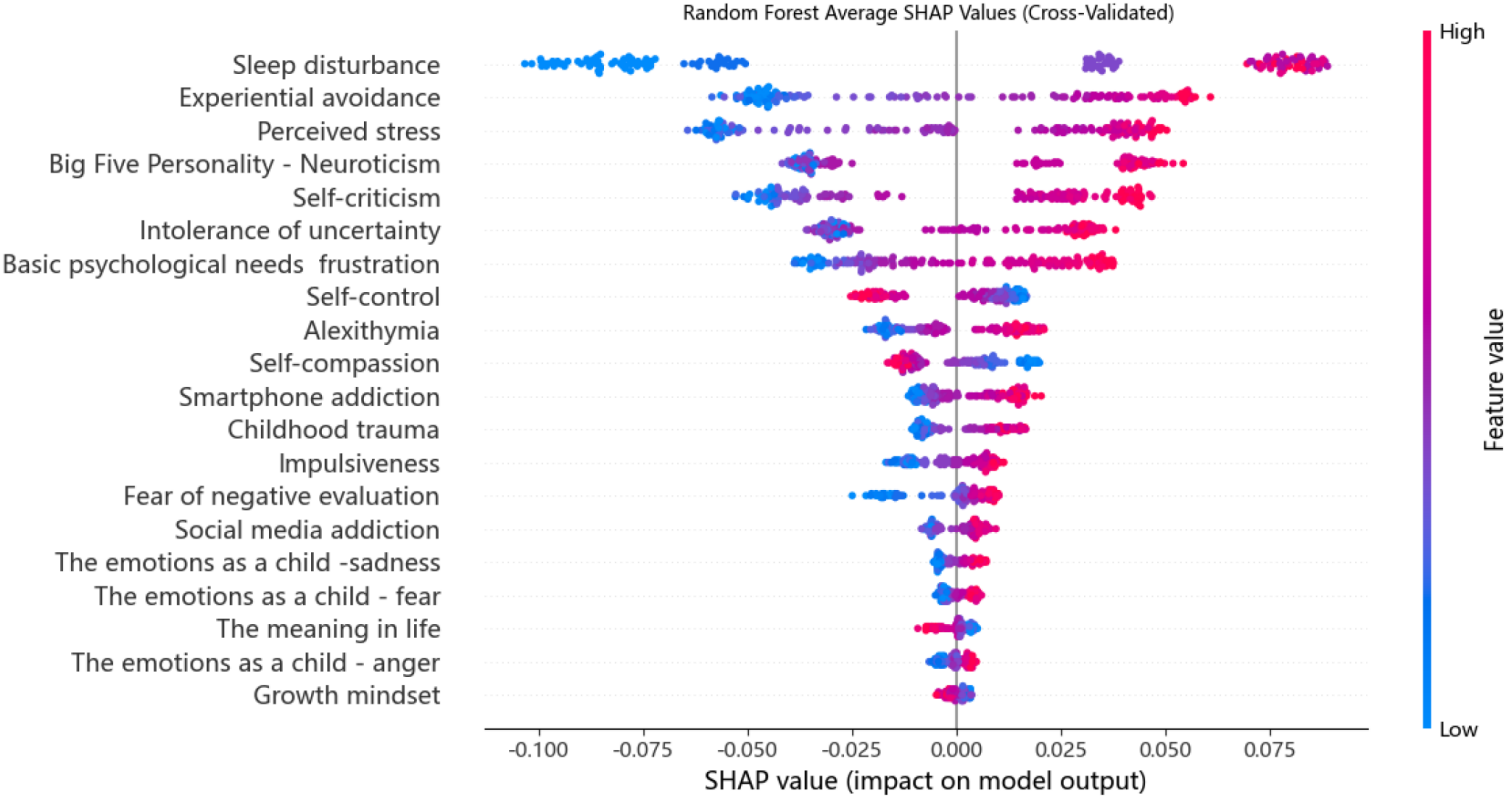
The summary plot of SHAP values for each feature of the Random Forest model.

To further analyze the contribution of each feature to depressive symptom prediction, SHAP analysis was conducted on the best-performing Random Forest model, with the results shown in **Figure 4**. In this visualization, higher SHAP values indicate greater impact on the model’s predictions. Each point represents a subject’s feature value, with color indicating value level (red: higher, bule: lower). Points are stacked vertically to indicate data density. Features with high positive SHAP values substantially increase the predicted probability of depressive symptoms, while features with negative SHAP values may reduce it. This visualization facilitates the interpretation of both the individual contribution and interactive role of each predictor within the model. Sleep disturbance had the greatest influence, followed by perceived stress, experiential avoidance, self-criticism, and frustration of basic psychological needs, with predictive influence progressively declining thereafter.

**Figure 5:**
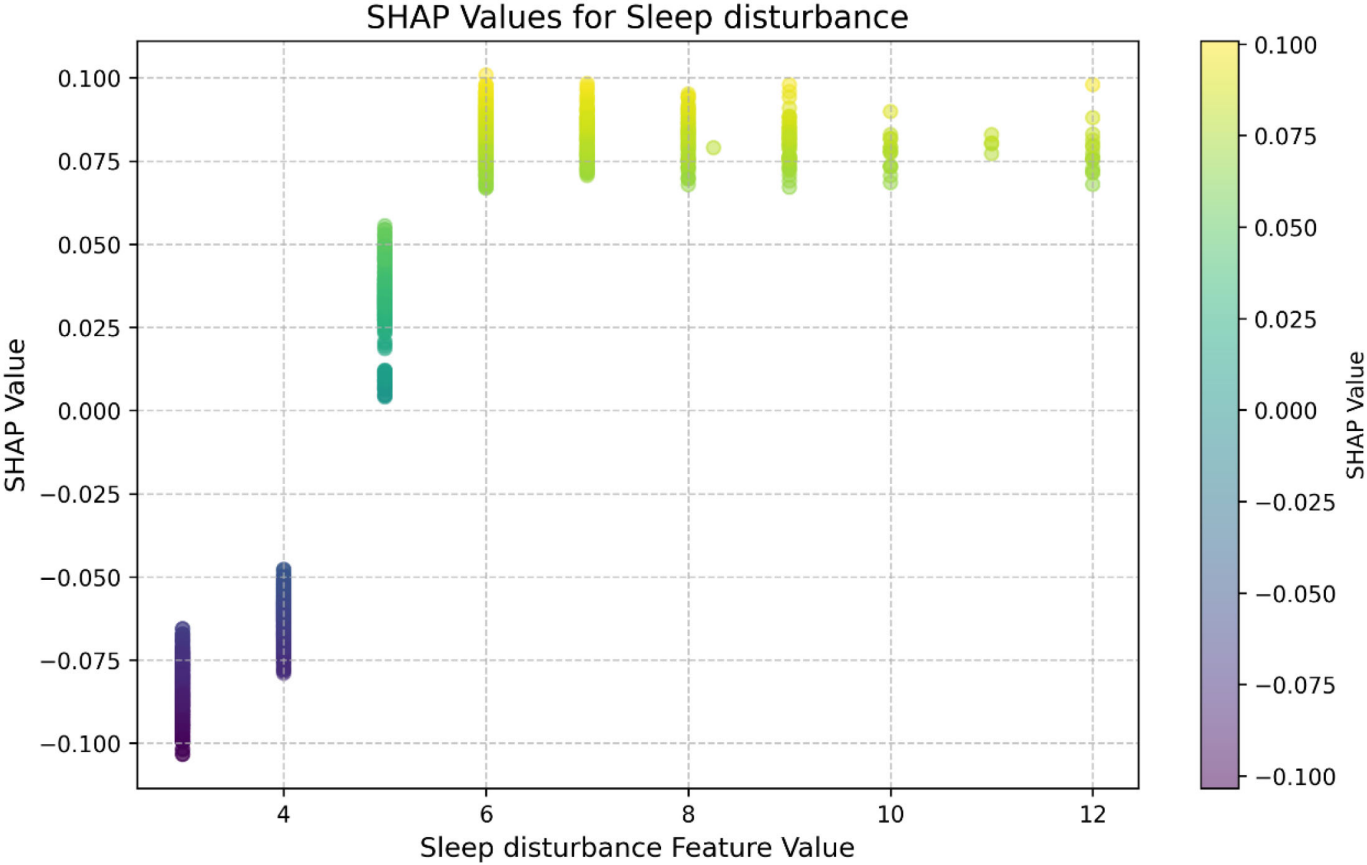
SHAP value plot of sleep disturbance varying with actual values.

Given the high predictive value of sleep disturbance shown in both the feature importance and SHAP value plots, we conducted a SHAP scatter plot analysis to further examine this relationship. **Figure 5** illustrates the association between the actual values of the sleep disturbance feature and their corresponding SHAP values. The plot shows that as sleep disturbance scores increase, SHAP values rise steadily within the range of 0 to 6. This suggests that greater sleep disturbance contributes positively to the model’s prediction of depressive symptoms. Between values of 6 and 12, the upward trend continues but becomes more variable, indicating possible instability or the influence of additional interacting factors at higher levels of sleep disturbance. Overall, sleep disturbance exerts a consistently positive effect on model predictions across its range, reinforcing its role as a key predictor of depressive symptoms.

## Discussion

### Prediction of depressive symptoms

In the current research, we developed and compared multiple ML models incorporating 38 psychosocial and demographic predictors to predict depressive symptoms among higher education students in China. Among the tested algorithms, the Random Forest model demonstrated superior performance, outperforming XGBoost, LightGBM, and SVM in terms of accuracy (0.7908), precision (0.7878), recall (0.8041), F1-score (0.7956), and AUC (0.8704). These results align with previous research (22), indicating that the model achieves high predictive accuracy and effectively identifies college students at elevated risk of depressive symptoms.

The superiority of the Random Forest model can be attributed to its unique learning mechanisms, which are well-suited to the complexity of the data used in this study. As an ensemble learning method, Random Forest enhances model stability and predictive accuracy by constructing multiple decision trees and aggregating their predictions through majority voting or averaging. This ensemble approach substantially reduces the risk of overfitting, a common limitation in traditional single-tree models. The dataset in this study included 38 predictive variables. Random Forest’s capacity to handle high-dimensional data and model complex feature interactions makes it particularly suitable for predicting depressive symptoms, which are influenced by numerous interrelated factors (66). Additionally, the algorithm introduces randomness during model construction by selecting a random subset of features at each split. This embedded feature selection not only improves computational efficiency but also helps reduce the impact of noisy variables (67).

In this study, although XGBoost and LightGBM are known for their effectiveness in handling imbalanced data and complex datasets, they underperformed compared to the Random Forest model. XGBoost, while efficient in large datasets, is sensitive to hyperparameter tuning and prone to overfitting in noisy data (56, 68). Similarly, LightGBM’s leaf-wise growth strategy improves computational efficiency but may lead to overfitting in smaller or imbalanced datasets (57). Although SVM is recognized for its generalization capability in high-dimensional, small-sample scenarios, it lagged behind Random Forest in terms of accuracy and AUC, likely due to its limited capacity to capture nonlinear relationships among predictors (58, 69).

Previous studies predicting depressive symptoms have rarely used cognitive and emotion-related variables (70, 71), but this study has used these variables, such as self-control, emotion regulation, meaning in life, growth mindset, Self-compassion, experience avoidance, and more. This gives us a glimpse into the mechanisms by which cognitive and emotional factors contribute to depressive symptoms. Moreover, while previous research has often focused on predicting depressive symptoms in children and adolescents (72, 73), this article focuses specifically on the university student population – a group highly susceptible to depressive symptoms – yielding valuable insights.

### Variable importance

Model interpretation was performed using the SHAP approach. SHAP is a personalized feature attribution method that strictly satisfies the Consistency axiom. This property is a core strength of its mathematical foundation (Shapley values from game theory). When a feature’s contribution to model prediction increases, its SHAP value must increase (or remain unchanged), ensuring no contradictory attributions (74). Therefore, the interpretability of our machine learning models meets the requirements for consistency. SHAP analysis reveals that predictors spanning multiple domains significantly contribute to forecasting depressive symptoms in Chinese university students, aligning with ecological systems theory (8).

First, the results suggest sleep disturbance emerged as the most influential contributor. This finding corroborates earlier studies that have found a close connection between sleep disturbances and depressive symptoms in university populations (75, 76). Shorter sleep duration exhibits a relationship with a higher risk of developing depressive disorders (77), and inadequate sleep hygiene may contribute to the development of depressive symptoms (78). Underlying these associations is the potential for neural dysregulation. Specifically, inadequate sleep disrupts dopamine activity in the limbic system and striatum, compromising the brain’s reward circuitry and increasing susceptibility to mental illness (79, 80). Additionally, substantial evidence suggests that sleep problems during adolescence can elevate depressive symptoms by impairing both cognitive performance and emotion regulation (81–83).

In addition, this study found that perceived stress was among the top four predictors of depressive symptoms. The present findings resonate with established research. For example, Leung et al. (84) identified perceived stress as a robust predictor of adolescent depression. Parallel results emerged in a Chinese student sample, where greater perceived stress predicted more severe depressive symptoms (85). We can explain it theoretically. Firstly, according to the stress–reward–mentalizing model of depression, the disorder can be conceptualized as a developmental, stress-related condition. When combined with heightened stress sensitivity, elevated stress significantly increases vulnerability to depressive symptoms (86). Additionally, evidence suggests that recent life stress may exacerbate underlying vulnerabilities in HPA axis functioning, with these risk factors potentially interacting synergistically to increase the likelihood of adolescent depression (87).

Experiential avoidance also emerges as a risk factor for depressive symptoms among college students in this study, consistent with findings from Núñez et al. (88). Experiential avoidance manifests as inflexible cognitive-behavioral patterns aimed at suppressing distressing intrapsychic content (89). Research has shown that experiential avoidance is a core transdiagnostic process underlying a range of psychological disorders, including depression, anxiety, and post-traumatic stress disorder (90). College students who engage in experiential avoidance are often unwilling to experience unpleasant emotions or thoughts and may actively suppress or avoid them. Paradoxically, attempts to avoid depressive thoughts can heighten their salience, ultimately intensifying depressive emotional experiences (91).

Self-criticism emerged as a key predictor of depressive symptoms in the current research. Cumulative research findings establish that self-criticism contributes to increased levels of depression and functions as both a risk factor and a perpetuating factor for depressive disorders (92, 93). Blatt (94) theorized that individuals with pronounced self-critical tendencies exhibit “intense feelings of inferiority, guilt, and worthlessness and by a sense that one has failed to live up to expectations and standards”. Research further suggests that individuals who frequently engage in self-critical thought patterns show greater susceptibility to fall into cycles of rumination and self-doubt, perpetuating ongoing emotional distress (95). These cognitive-emotional patterns are strongly associated with heightened vulnerability to depressive symptoms (94). Among university students, self-criticism often arises from the internalized pressure to meet excessively high personal expectations, thereby increasing their susceptibility to depression and related mental health concerns (96).

### Strengths, limitations, and future directions

This study has several limitations. First, the sample size was relatively small; expanding the sample in future research would improve the model’s generalizability. Second, participants were drawn from only six universities located in central and southern China, which may not fully capture regional differences, particularly those between urban and rural areas or between eastern and western regions of the country. For example, disparities in access to mental health resources among rural students may influence the prevalence or severity of depressive symptoms. Third, the current models included only sociodemographic and psychosocial variables, lacking objective physiological indicators such as cortisol concentration or EEG alpha wave power. As demonstrated by Wollenhaupt-Aguiar et al. (97), combining ML with peripheral biomarker measurements can produce more objective and robust predictive features. Future research should consider integrating physiological indicators to further enrich the comprehensiveness and accuracy of depressive symptom prediction among college students.

Although limited in some aspects, this study demonstrates meaningful strengths with important implications. First, it constructed a predictive model based on Bronfenbrenner’s ecosystem theory using ML techniques, incorporating 38 predictors across multiple levels simultaneously, which has stronger explanatory power than traditional regression models. Second, multiple models were used to systematically compare the predictive performance of Random Forest, XGBoost, LightGBM, and SVM for depression symptoms among Chinese university students, overcoming the limitations of single-algorithm research. Finally, through feature importance and SHAP analyses, the factors influencing depressive symptoms among college students were ranked, and several key influencing factors were identified. Analyzing the conditions of college students in these areas can help to promptly identify those at risk of developing depressive symptoms and support more targeted, data-informed interventions, potentially offering a more accurate and efficient approach than standard PHQ-9 screening.

## Conclusion

This study developed and compared multiple ML models to predict depressive symptoms among Chinese college students, incorporating 38 psychosocial and demographic predictors spanning individual, familial, and social domains. Among the models tested, the Random Forest algorithm demonstrated superior performance, outperforming XGBoost, LightGBM, and SVM in integrating complex predictors. Sleep disturbance, perceived stress, experiential avoidance, and self-criticism emerged as the most robust predictors of depressive symptoms. These findings underscore the utility of ML in synthesizing multidimensional factors to support the early identification of high-risk individuals and inform targeted mental health interventions. Such approaches can assist schools and healthcare systems in implementing proactive, personalized mental health strategies, ultimately contributing to efforts to reduce the growing burden of depression among college students.

## Data Availability

The raw data supporting the conclusions of this article will be made available by the authors, without undue reservation.
