# Supplementary material for: Machine learning models for predicting the risk of depressive symptoms in Chinese college students
Source: Front Psychiatry. 2025 Aug 5;16:1648585. doi: 10.3389/fpsyt.2025.1648585 (PMC12361154; doi:10.3389/fpsyt.2025.1648585)
Supplement: Supplementary file 1 [file Supplementaryfile1.docx]

**Online Supplementary Materials for**

**Machine Learning Models for Predicting the Risk of Depressive Symptoms in Chinese College Students**

**Section 1**

Detailed information about the measures of the predictor variables for depressive symptoms is presented below:

**Smartphone Usage Time**
Daily smartphone usage was assessed through a single item (e.g., “In the past six months, how many hours on average did you spend using your smartphone each day?”). Responses were recorded on a 7-point Likert scale from 1 (*no usage*) to 7 (*more than 10 hours*).

**Pre-sleep Smartphone Usage**
Evening smartphone use was measured with one item (e.g., “In the past six months, how long did you typically use your smartphone before bedtime?”). Responses were recorded on a 7-point Likert scale from 1 (*not at all*) to 7 (*more than 2 hours*).

**Academic Stress**
Perceived academic stress was evaluated using a single-item measure (e.g., “How would you describe your academic stress level?”). Participants rated their stress on a 5-point Likert scale from 1 (*no stress*) to 5 (*extremely high stress*).

**Employment Pressure**
Career-related pressure was assessed with one item (e.g., “How much pressure do you feel regarding future employment/education?”). Responses ranged from 1 (*no pressure*) to 5 (*very high pressure*) on a 5-point Likert scale.

**Alexithymia**

The Perth Alexithymia Questionnaire-Short Form, developed by Preece et al. (2023), was used to measure alexithymia. The scale consists of three factors: difficulty identifying feelings, difficulty describing feelings, and externally oriented thinking, totaling 6 items (e.g., “I tend to ignore how I feel”). A seven-point Likert scale is used (1 = *strongly disagree*, 7 = *strongly agree*), with higher scores indicating more pronounced alexithymia characteristics. The Cronbach’s α in this study was 0.86.

**Sleep Disturbance**

The study evaluated sleep-related difficulties using a three-item subset from the German adaptation of the Symptom-Checkliste-90-R inventory (Schmitz et al., 2000). Participants rated their experiences with sleep onset problems, premature awakening, and restless sleep patterns during the previous week, using a four-point scale ranging from 1 (*not at all*) to 4 (*extreme*). An example item includes, “Difficulty falling asleep.” These indicators were combined to form a composite measure representing general sleep disturbance. These items have been successfully employed in previous research (Nguyen et al., 2023). The Cronbach’s α in this study was 0.74.

**Suicidal Ideation, Suicide Plans, Suicide Attempts**

The study used a three-item questionnaire (Centers for Disease Control and Prevention, 2013) to measure suicidal ideation (e.g., “Have you ever thought about killing yourself during the past year?”), suicide plans (e.g., “Have you ever made a specific plan about how to kill yourself during the past year?”), and suicide attempts (e.g., “Have you ever tried to kill yourself during the past year?”). All items were rated on a two-point scale (0 = *never*, 1 = *yes*).

**Non-Suicidal Self-Injury**

The study assessed seven common non-suicidal self-injury behaviors in the past year (Gong et al., 2019). An example item is “In the past 6 months, have you deliberately cut yourself but without suicidal intent?” All the seven NSSI behavior items were rated on a four-point scale, ranging from 0 (*never*) to 3 (*six or more*). Higher scores indicated greater severity of non-suicidal self-injury. The Cronbach’s α in this study was 0.86.

**Smartphone Addiction**

Smartphone addiction was measured using the short version of the Smartphone Addiction Scale developed by Kwon et al. (2013), comprising a final selection of 10 items (e.g., “Missing planned work due to smartphone use”). A six-point Likert scale ranging from 1 (*strongly disagree*) to 6 (*strongly agree*) was utilized, with higher scores indicating more severe smartphone addiction. The Cronbach’s α in this study was 0.86.

**Social Media Addiction**

The Bergen Social Media Addiction Scale, formulated by Leung et al. (2019), was employed to measure social media addiction. It encompasses six items assessing salience, mood modification, tolerance, withdrawal symptoms, conflict, and relapse (e.g., “Felt an urge to use social media more and more?”). Scoring is conducted on a five-point Likert scale (1 = *very rarely*, 5 = *very often*), with higher scores denoting greater degrees of social media addiction. The Cronbach’s α in this study was 0.76.

**Self-Control**

The Brief Self-Control Scale, revised by Morean et al. (2014) and introduced to China by Luo et al. (2021), was employed to measure self-control. The scale includes two subscales: self-discipline and impulse control, with a total of 7 items (e.g., “I am good at resisting temptation”). Using a five-point Likert scale (1 = *completely inconsistent*, 5 = *completely consistent*), higher scores reflect greater levels of self-control. The Cronbach’s α in this study was 0.67.

**Emotion Regulation**

The Emotion Regulation Scale, developed by Gross & John (2003) and adapted into Chinese by Wang (2007), was employed to measure emotion regulation. It contains cognitive reappraisal and expressive suppression. An example item includes, “I control my emotions by not expressing them.” Scores are assigned on a seven-point scale (1 =*strongly disagree*, 7 = *strongly agree*), with higher scores indicating more frequent use emotion regulation strategy. The Cronbach’s α in this study was 0.79.

**Growth Mindset**

The Growth Mindset Scale (Dweck, 2006) consists of two dimensions: fixed mindset and growth mindset, with six items in total. The fixed mindset dimension includes three items (e.g., “Intelligence is difficult to change”), designed to assess participants’ tendencies toward a fixed mindset. The growth mindset dimension includes three items (e.g., “No matter who you are, you can change your intelligence to a great extent”), designed to assess participants’ tendencies toward a growth mindset. The scale employs a six-point scale, from 1 (*strongly disagree*) to 6 (*strongly agree*). The fixed mindset items were reverse-scored. A higher score indicates a stronger tendency toward a growth mindset, whereas a lower score indicates a stronger tendency toward a fixed mindset. In this study, the scale demonstrated good reliability, with a Cronbach’s α coefficient of 0.85.

**Self-Compassion**

The Self-Compassion Scale-Short Form, developed by Neff (2003) and translated into Chinese by Gong et al. (2014), was employed to measure self-compassion. The scale consists of three dimensions: self-kindness, common humanity, and mindfulness. The scale comprises 12 items (e.g., “When something painful happens I try to take a balanced view of the situation”), which were rated from 1 (*never*) to 5 (*always*),. Higher scores indicating greater levels of self-compassion. The Cronbach’s α in this study was 0.76.

**Meaning in Life**

The Chinese Revised Version of the Meaning in Life Questionnaire was employed to measure students’ sense of life meaning (Wang, 2013). The scale consists of 10 items and is divided into two subscales: search for meaning (5 items, e.g., “I am searching for the meaning of my life”) and presence of meaning (5 items, e.g., “My life has a clear direction”). Each item is rated on a seven-point scale, ranging from 1 (*completely disagree*) to 7 (*completely agree*). Higher scores indicating greater levels of meaning in life. In this study, the Cronbach’s α was 0.90.

**Fear of Negative Evaluation**

The Chinese version of the Fear of Negative Evaluation Scale, developed by Watson and Friend (1969) and adapted by Chen (2002), was used to assess fear of negative evaluations. The scale consists of 12 items (e.g., “I am afraid when others are dissatisfied with me”), which were rated from 1 (*completely inconsistent*) to 5 (*completely consistent*). In this survey, the Cronbach’s α was 0.86.

**Self-Criticism**

The self-criticism subscale of the Depressive Experiences Questionnaire (Bagby et al., 1994), which consists of 9 items (e.g., “Many times I feel helpless”), was employed to measure self-criticism. The scale uses a five-point Likert scale ranging from 1 (*completely inconsistent*) to 5 (*completely consistent*). Higher scores indicating greater levels of self-criticism. The Cronbach’s α in this study was 0.88.

**Basic psychological needs frustration**

The basic psychological need frustration subscale from the Basic Psychological Need Satisfaction and Frustration Scale (Chen et al., 2015) was used to assess of psychological needs frustration. The scale comprised 12 items (e.g., “I feel pressured to do too many things”). All items were rated on a five-point Likert scale ranging from 1 (*completely inconsistent*) to 5 (*completely consistent*). The Cronbach’s α in this study was 0.91.

**Impulsivity**

The Brief Barratt Impulsiveness Scale, developed by Morean et al. (2014) and translated and revised by Luo et al. (2020a), was used to measure impulsivity. The 8-item scale are scored on a four-point Likert scale (1 = *never*, 4 = *always*). An example item includes, “I plan tasks carefully.” Higher mean scores indicate greater impulsivity among participants. The Cronbach’s α in this study was 0.74.

**Experiential Avoidance**

The Acceptance and Action Questionnaire, developed by Bond et al. (2011) and revised by Cao et al. (2013), was used to measure experiential avoidance. The 7-item scale are scored on a seven-point scale (1 = *never true*, 7 = *always true*). An example item includes, “Emotions cause problems in my life” Higher scores denote increased experiential avoidance. The Cronbach’s α in this study was 0.93.

**Intolerance of Uncertainty**

Brief Intolerance of Uncertainty Scale, originally developed by Carleton et al. (2007) and revised for Chinese middle school students by Wu et al. (2016), was used to measure intolerance of uncertainty. This scale consists of 12 items (e.g., “I must get away from all uncertain situations”), which were rated from 1 (*completely inconsistent*) to 5 (*completely consistent*). Higher cumulative scores indicating greater levels of intolerance of uncertainty. The Cronbach’s α in this study was 0.90.

**Big Five Personality Inventory**

The Chinese Big Five Personality Inventory-15 was used to measure five personality traits (Zhang et al., 2019). The scale used a 6-point scale ranging from 1 (*completely inconsistent*) to 6 (*completely consistent*). Specifically, three items measure neuroticism (e.g., “I often worry about trifles”); three items assess conscientiousness (e.g., “I like to plan things from the beginning”); three items evaluate agreeableness (e.g., “I think most people are well-intentioned”); three items measure openness (e.g., “I like adventure”); and three items assess extraversion (e.g., “I like to go to social and recreational parties”). In the present study, the Cronbach’s α for these five dimensions were 0.84, 0.69, 0.81, 0.87, and 0.78, respectively.

**Childhood Trauma**

This study utilized the emotional abuse, physical abuse, emotional neglect, and physical neglect subscales of the Childhood Trauma Questionnaire to measure childhood trauma (Bernstein et al., 2003), including 23 items (e.g., “Someone tried to touch me in a sexual way or made me touch them”). The scale are scored on a five-point Likert scale ranging from 1 (*never*) to 5 (*always*). Higher total scores indicate more severe childhood trauma. The Cronbach’s α in this study was 0.86.

**Bullying Victimization**

The study used Bullying Victimization Questionnaire to measure the frequency of four specific types of bullying victimization: physical, verbal, social/relational, and cyberbullying (Chao et al., 2023). This scale consists of 4 items (e.g., “How many times have you been physically bullied in the last year?”), which were rated from 1 (*never*) to 5 (*seven or more*) Higher total scores indicate more bullying victimization. Cronbach’s α was 0.70.

**Perceived Stress**

The Perceived Stress Scale, developed by Cohen et al. (1983) and translated by Yang and Huang (2003), was employed in this study. The scale consists of 14 items rated on a 5-point Likert scale (1 = *never* to 5 = *always*), with higher total scores indicating greater levels of perceived stress. An example item includes, “In the last month, how often have you felt that things were going your way?” The Cronbach’s α in this study was 0.80.

**Parental emotion socialization**

The Emotions as a Child Scale (EAC)--Chinese Version (Luo et al., 2020b) was adapted from the 45-item EAC (Magai & O’Neal, 1997) to assess parental emotion socialization for three negative emotions—anger, fear, and sadness. The revised EAC consists of 36 items. The scale includes an anger subscale with 12 items (e.g., "When I was angry, my parents told me not to be angry"), a fear subscale with 12 items (e.g., "When I felt scared, my parents helped me solve the problem"), and a sadness subscale with 12 items (e.g., "When I felt sad, my parents told me not to worry"). Each subscale of the EAC comprises two dimensions: supportive strategies and non-supportive strategies. Each item is scored on a 5-point Likert scale ranging from 1 ("Never") to 5 ("Always"). Higher scores on each dimension indicate a greater parental tendency to employ that particular emotion socialization strategy. In the current survey, the Cronbach’s α for the supportive strategies and non-supportive strategies dimensions were 0.89 and 0.84, respectively.

**Section 2**

Feature importance plots for XGBoost and LightGBM, along with SHAP plots for XGBoost, LightGBM, and SVM, provide insights into model behavior and feature contributions
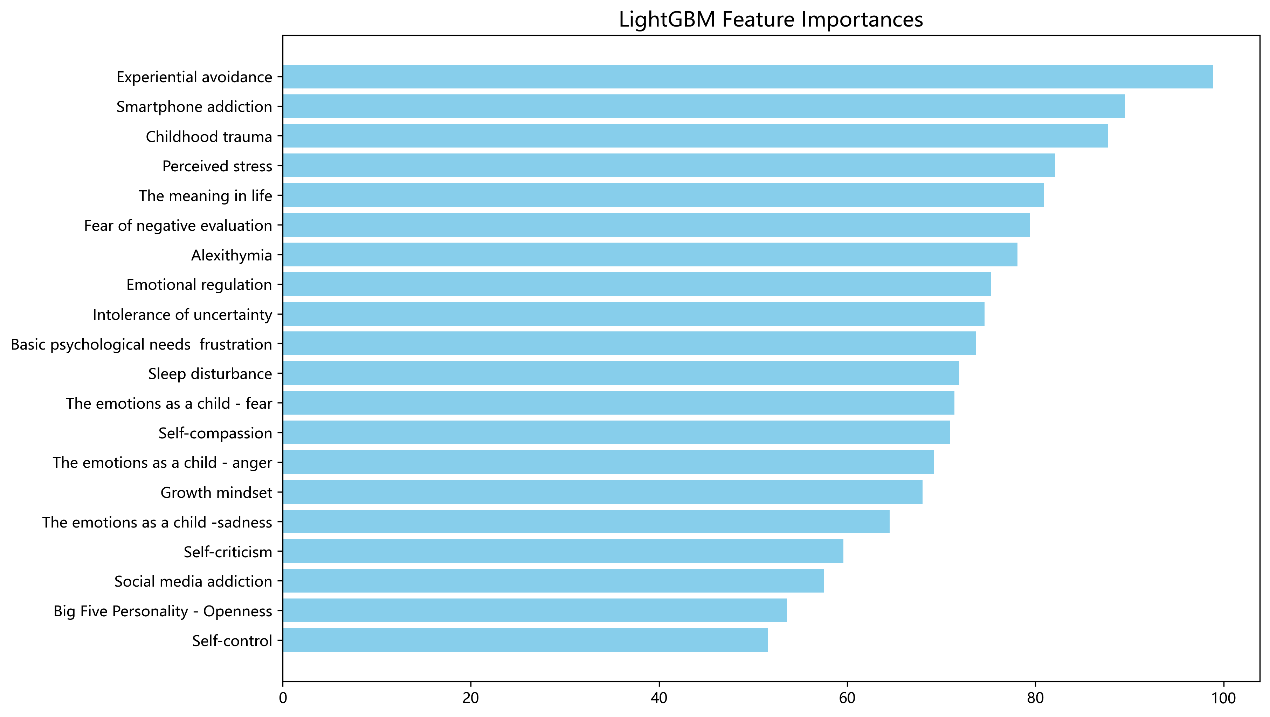


**Fig. 1S** Feature importance plot of the LightGBM, which depicts the importance of each variate in the development of the model.


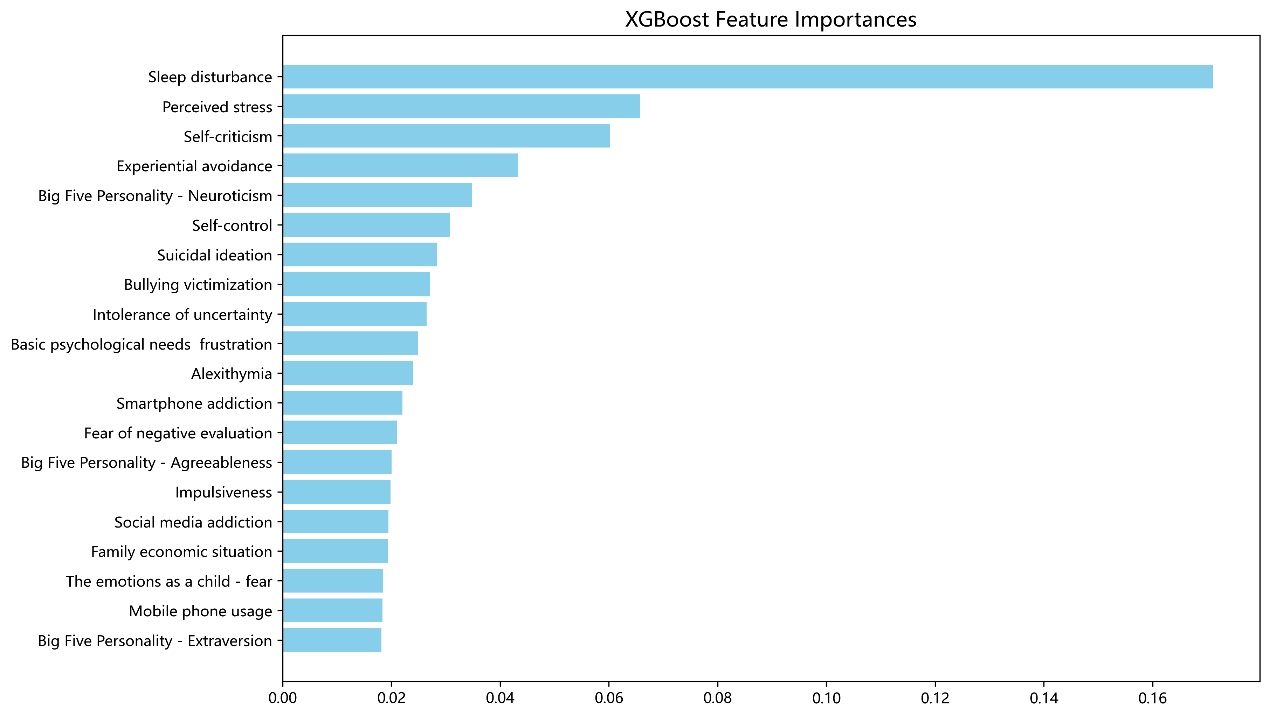


**Fig. 2S** Feature importance plot of the XGBoost, which depicts the importance of each variate in the development of the model.


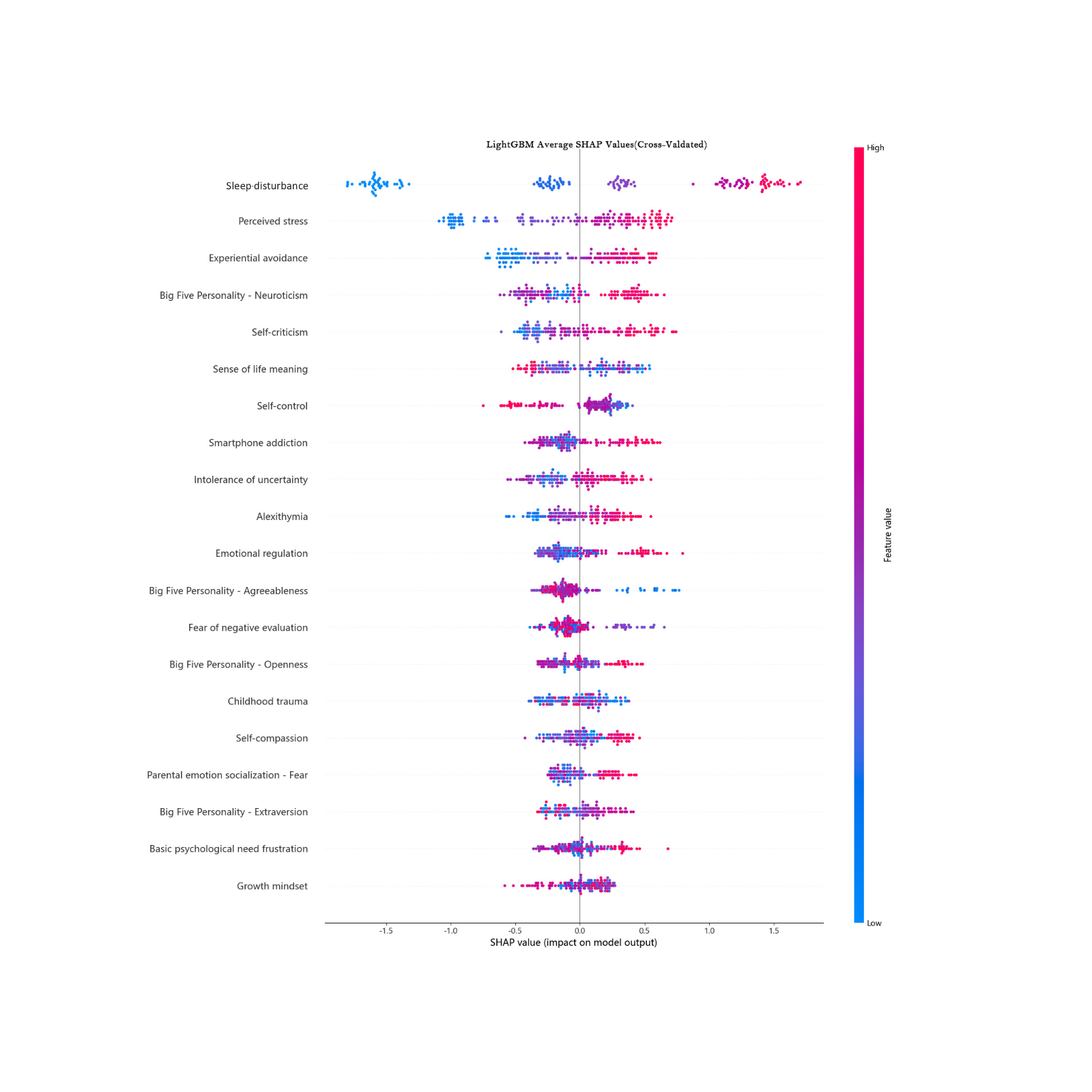


**Fig. 3S** The summary plot of SHAP values for each feature of the LightGBM model.


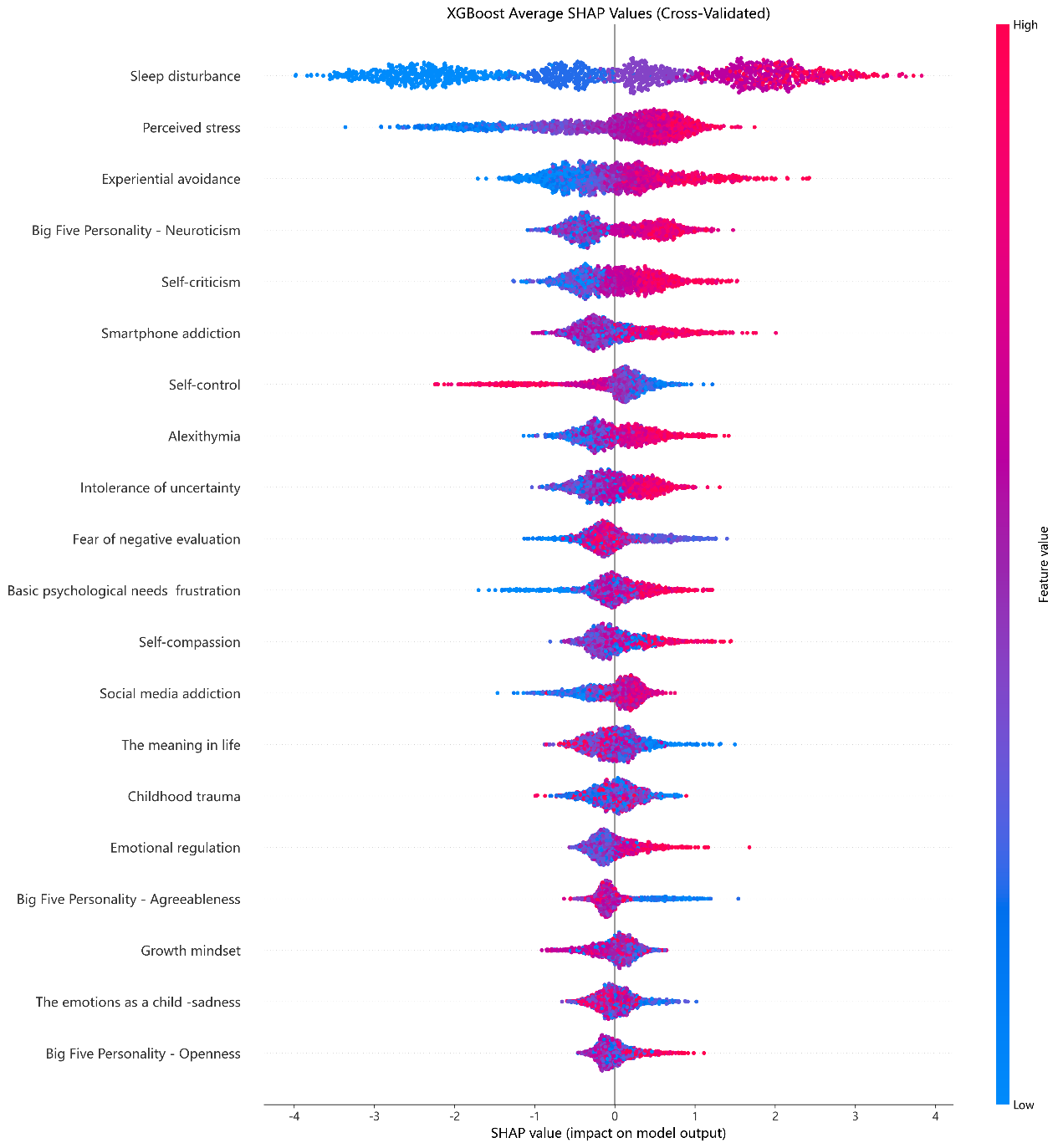


**Fig. 4S** The summary plot of SHAP values for each feature of the XGBoost model.

\


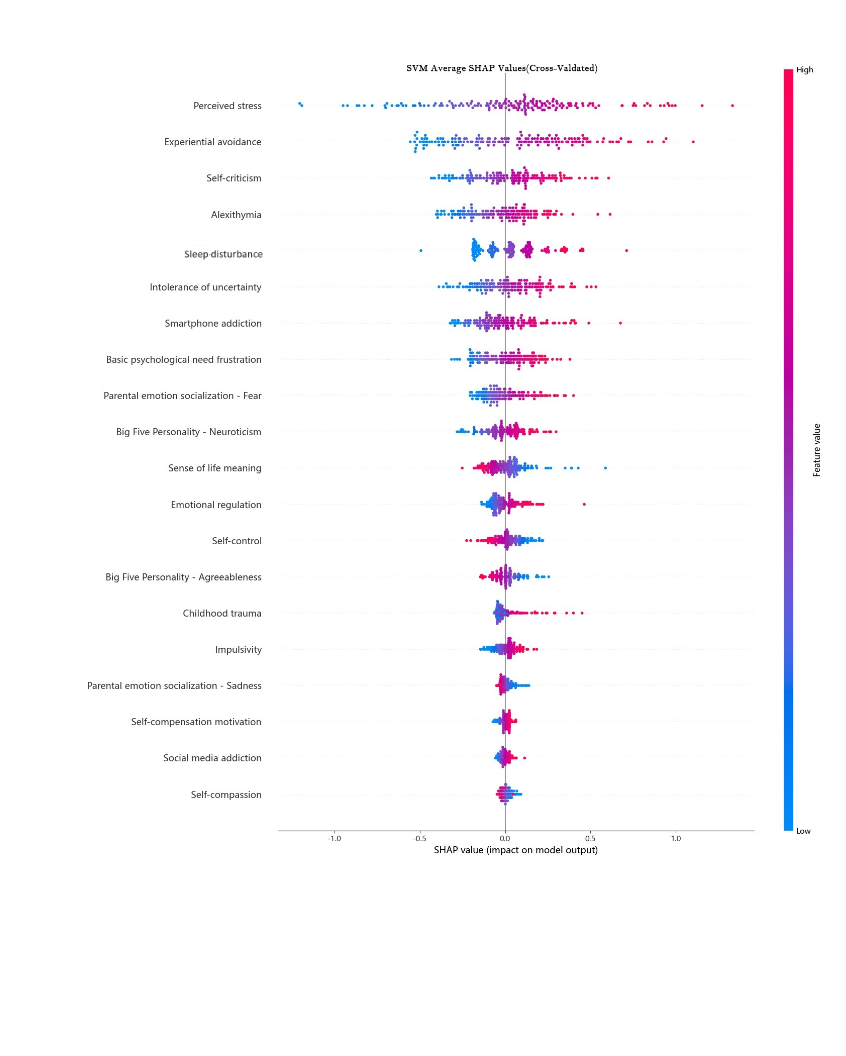


**Fig. 5S** The summary plot of SHAP values for each feature of the SVM model.

Among the four machine learning models established in this study, only the random forest model underwent hyperparameter optimization. The other three models were built using a random seed of random_state=57. The hyperparameters of the optimized random forest model are as follows: n_estimators=207, max_depth=4, min_samples_split=2, max_features=5, min_samples_leaf=50, random_state=57, and bootstrap=True.

**References**

Bagby, R. M., Parker, J. D., Joffe, R. T., & Buis, T. (1994). Reconstruction and validation of the Depressive Experiences Questionnaire. *Assessment*, *1*(1), 59–68. <https://doi.org/10.1177/1073191194001001009>

Bernstein, D. P., Stein, J. A., Newcomb, M. D., Walker, E., Pogge, D., Ahluvalia, T., Stokes, J., Handelsman, L., Medrano, M., Desmond, D., & Zule, W. (2003). Development and validation of a brief screening version of the Childhood Trauma Questionnaire. *Child Abuse & Neglect*, *27*(2), 169–190. <https://doi.org/10.1016/S0145-2134(02)00541-0>

Bond, F. W., Hayes, S. C., Baer, R. A., Carpenter, K. M., Guenole, N., Orcutt, H. K., Waltz, T., & Zettle, R. D. (2011). Preliminary Psychometric Properties of the Acceptance and Action Questionnaire–II: A Revised Measure of Psychological Inflexibility and Experiential Avoidance. *Behavior Therapy*, *42*(4), 676–688. <https://doi.org/10.1016/j.beth.2011.03.007>

Cao, J., Ji, Y., & Zhu, Z. (2013). Reliability and validity of the Chinese version of the Acceptance and Action Questionnaire-Second Edition (AAQ-Ⅱ) in college students. *Chinese Mental Health Journal*, *27* (11), 873-877. <https://doi.org/10.3969/j.issn.1000-6729.2013.11.014>

Carleton, R. N., Norton, M. A. P. J., & Asmundson, G. J. G. (2007). Fearing the unknown: A short version of the Intolerance of Uncertainty Scale. *Journal of Anxiety Disorders*, *21*(1), 105–117. https://doi.org/10.1016/j.janxdis.2006.03.014

Centers for Disease Control and Prevention. (2013). Adolescent and School Health: YRBSS Questionnaire 2013. CDC.

Chao, M., Lei, J., He, R., Jiang, Y., & Yang, H. (2023). TikTok use and psychosocial factors among adolescents: Comparisons of non-users, moderate users, and addictive users. *Psychiatry Research*, *325*, 115247. <https://doi.org/10.1016/j.psychres.2023.115247>

Chen, B., Vansteenkiste, M., Beyers, W., Boone, L., Deci, E. L., Van Der Kaap-Deeder, J., Duriez, B., Lens, W., Matos, L., Mouratidis, A., Ryan, R. M., Sheldon, K. M., Soenens, B., Van Petegem, S., & Verstuyf, J. (2015). Basic psychological need satisfaction, need frustration, and need strength across four cultures. *Motivation and Emotion*, *39*(2), 216–236. <https://doi.org/10.1007/s11031-014-9450-1>

Chen, Z. (2002). Fear of Negative Evaluation and Test Anxiety in Middle School Students. *Chinese Mental Health Journal*, *12*, 855–857. <https://doi.org/10.3321/j.issn:1000-6729.2002.12.020>

Cohen, S., Kamarck, T., & Mermelstein, R. (1983). A Global Measure of Perceived Stress. *Journal of Health and Social Behavior*, *24*(4), 385. <https://doi.org/10.2307/2136404>

Dweck, C. S. (2006). Mindset: The new psychology of success. Random house.

Gong, H., Jia, H., Guo, T., & Zou, L. (2014). Revision and Psychometric Validation of the Self-Compassion Scale for Adolescents. *Psychological Research*, *7*(1), 36–40, 79.

Gong, T., Ren, Y., Wu, J., Jiang, Y., Hu, W., & You, J. (2019). The associations among self-criticism, hopelessness, rumination, and NSSI in adolescents: A moderated mediation model. *Journal of Adolescence*, *72*, 1–9. <https://doi.org/10.1016/j.adolescence.2019.01.007>

Gross, J. J., & John, O. P. (2003). Individual differences in two emotion regulation processes: Implications for affect, relationships, and well-being. *Journal of Personality and Social Psychology*, *85*(2), 348–362. <https://doi.org/10.1037/0022-3514.85.2.348>

Kwon, M., Kim, D.-J., Cho, H., & Yang, S. (2013). The Smartphone Addiction Scale: Development and validation of a short version for adolescents. *PLoS ONE*, *8*(12), e83558. <https://doi.org/10.1371/journal.pone.0083558>

Leung, H., Pakpour, A. H., Strong, C., Lin, Y., Tsai, M., Griffiths, M. D., Lin, C., & Chen, I. (2019). Measurement invariance across young adults from Hong Kong and Taiwan among three internet-related addiction scales: Bergen Social Media Addiction Scale (BSMAS), Smartphone Application-Based Addiction Scale (SABAS), and Internet Gaming Disorder Scale-Short Form (IGDS-SF9) (Study Part A). *Addictive Behaviors*, *101*, 105969. <https://doi.org/10.1016/j.addbeh.2019.04.027>

Luo, J., Wang, M.-C., Gao, Y., Deng, J., & Qi, S.-S. (2020b). Factor structure and construct validity of the Emotions as a Child Scale (EAC) in Chinese children. *Psychological Assessment*, *32*(1), 85–97. <https://doi.org/10.1037/pas0000762>

Luo, T., Chen, L., Qin, L., & Xiao, S. (2021). Reliability and validity of Chinese Version of Brief Self-Control Scale. *Chinese Journal of Clinical Psychology*, *29*(1), 83–86. <https://doi.org/10.16128/j.cnki.1005-3611.2021.01.017>

Luo, T., Chen, M., Ouyang, F., & Xiao, S. (2020a). Reliability and validity of Chinese Version of Brief Barratt Impulsiveness Scale. *Chinese Journal of Clinical Psychology*, *28*(6), 1199–1201, 1280. <https://doi.org/10.16128/j.cnki.1005-3611.2020.06.025>

Magai, C., & O’Neal, C. R. (1997). Emotions as a child (child version). Unpublished manuscript, New York: Long Island University, Brooklyn.

Morean, M. E., DeMartini, K. S., Leeman, R. F., Pearlson, G. D., Anticevic, A., Krishnan-Sarin, S., Krystal, J. H., & O’Malley, S. S. (2014). Psychometrically improved, abbreviated versions of three classic measures of impulsivity and self-control. *Psychological Assessment*, *26*(3), 1003–1020. <https://doi.org/10.1037/pas0000003>

Neff, K. (2003). Self-compassion: An alternative conceptualization of a healthy attitude toward oneself. *Self and Identity*, *2*(2), 85–101. <https://doi.org/10.1080/15298860309032>

Nguyen, T. P., Lerch, S., Maggetti, A., Reichl, C., Tarokh, L., & Kaess, M. (2023). The relationship between sleep disturbance and self-harming behaviours in high-risk clinical adolescents. *Journal of Psychiatric Research*, *158*, 81–87. <https://doi.org/10.1016/j.jpsychires.2022.12.034>

Preece, D. A., Mehta, A., Petrova, K., Sikka, P., Bjureberg, J., Chen, W., Becerra, R., Allan, A., Robinson, K., & Gross, J. J. (2023). The Perth Alexithymia Questionnaire-Short Form (PAQ-S): A 6-item measure of alexithymia. *Journal of Affective Disorders*, *325*, 493–501. <https://doi.org/10.1016/j.jad.2023.01.036>

Schmitz, N., Hartkamp, N., Kiuse, J., Franke, G. H., Reister, G., & Tress, W. (2000). The symptom check-list-90-R (SCL-90-R): A German validation study. *Quality of Life Research*, *9*, 185–193.

Wang, L., Liu, H., Li, Z., & Du, W. (2007). Reliability and Validity of Emotion Regulation Questionnaire-Chinese Revised Version (ERQ-CRV). *China Journal of Health Psychology*, *6*, 503–505. <https://doi.org/10.13342/j.cnki.cjhp.2007.06.012>

Wang, X. (2013). Psychometric Evaluation of the Meaning in Life Questionnaire in Chinese Middle School Students. *Chinese Journal of Clinical Psychology*, *21*(5), 764–767+763. <https://doi.org/10.16128/j.cnki.1005-3611.2013.05.008>

Watson, D., & Friend, R. (1969). Measurement of social-evaluative anxiety. *Journal of Consulting and Clinical Psychology*, *33*(4), 448–457. <https://doi.org/10.1037/h0027806>

Wu, L., Wang, J., & Qi, X. (2016). Validity and reliability of the Intolerance of Uncertainty Scale-12 in middle school students. *Chinese Mental Health Journal*, *30*(9), 700–705. <https://doi.org/10.3969/j.issn.1000-6729.2016.09.012>

Yang, T., & Huang, H. (2003). An epidemiological study on stress among urban residents in social transition period. *Chinese Journal of Epidemiology*, *9*, 11–15.

Zhang, X., Wang, M., He, L., Jie, L., & Deng, J. (2019). The development and psychometric evaluation of the Chinese Big Five Personality Inventory-15. *PLoS ONE*, *14*(8), e0221621. <https://doi.org/10.1371/journal.pone.0221621>
